# Characterization of Flexible Copolymer Optical Fibers for Force Sensing Applications

**DOI:** 10.3390/s130911956

**Published:** 2013-09-09

**Authors:** Marek Krehel, René M. Rossi, Gian-Luca Bona, Lukas J. Scherer

**Affiliations:** 1 Empa, Swiss Federal Laboratories for Materials Science and Technology, Laboratory for Protection and Physiology, Lerchenfeldstrasse 5, St. Gallen 9014, Switzerland; E-Mails: marek.krehel@empa.ch (M.K.); rene.rossi@empa.ch (R.M.R.); gian-luca.bona@empa.ch (G.-L.B.); 2 ETH Zurich, Swiss Federal Institute of Technology, Department of Information Technology and Electrical Engineering, Gloriastrasse 35, Zurich 8092, Switzerland

**Keywords:** force sensor, optical fibres, sensitive force sensor, flexible light guide

## Abstract

In this paper, different polymer optical fibres for applications in force sensing systems in textile fabrics are reported. The proposed method is based on the deflection of the light in fibre waveguides. Applying a force on the fibre changes the geometry and affects the wave guiding properties and hence induces light loss in the optical fibre. Fibres out of three different elastic and transparent copolymer materials were successfully produced and tested. Moreover, the influence of the diameter on the sensing properties was studied. The detectable force ranges from 0.05 N to 40 N (applied on 3 cm of fibre length), which can be regulated with the material and the diameter of the fibre. The detected signal loss varied from 0.6% to 78.3%. The fibres have attenuation parameters between 0.16–0.25 dB/cm at 652 nm. We show that the cross-sensitivies to temperature, strain and bends are low. Moreover, the high yield strength (0.0039–0.0054 GPa) and flexibility make these fibres very attractive candidates for integration into textiles to form wearable sensors, medical textiles or even computing systems.

## 1. Introduction

Optical fibres-based sensors have numerous advantages compared to electrical or chemical devices, e.g., insensitivity to electromagnetic fields, water and corrosion resistance, compact size and light weight [1,2]. Due to these benefits, optical fibre based sensors are used in numerous applications to detect changes in chemical and/or physical parameters [1,3–6]. Various methods to detect pressure using optical fibres are known. Wang *et al.* proposed a pressure sensor that employs the effect of photoelasticity [7]. Urban *et al.* designed a pressure sensor that relies on FBG lateral deformation [8]. Within the Seventh Framework Programme “Ofseth”, optical fibres using optical time-domain reflectometry for distributed respiration measurement and optical fibres with long period gratings in microstructured POF were incorporated into elastic fabrics to measure the breathing rate and the breathing volume by measuring the changes in applied pressure [9–11]. The most common method to determine the pressure using optical fibres is by using fibre Bragg gratings [8,12–14]. To implement optical fibre based pressure sensors into textile fabrics, the fibres should be highly flexible, which is not the case for glass-based fibres most often used for fibre Bragg grating based sensors. Moreover, sensors based on fibre Bragg gratings have to be single mode and are dependent on temperature, strain and pressure simultaneously and require compensation of some of the parameters in order to sense one of them [15,16].

In this study, elastically deformable light pipes, so called multimode optical fibres which react to applied pressure by deflecting light in the fibre structure, are reported. Due to their high flexibility and their appropriate tensile strength (from 0.0033 to 0.0056 GPa), they meet the critical criteria to form medical textiles [17,18]. Long-term body monitoring solutions using textile fabrics have been intensively studied in recent years [19,20]. Plastic optical fibres have several advantages compared to textile electronics when wearing them close to the body, e.g., comfort, ease of movements, and reduced movement artefacts [17]. The electronics involved in the proposed solutions often influence negatively the haptic of medical textiles. Flexible and smart fibres allow the separation of the rigid electronics from the measured region and thus from the body. Possible body parameters which could be measured with the proposed solution are, e.g., muscles activity, motion detections or breath monitoring [21]. For technical applications, these pressure sensors can be used for counting occupied seats in airplanes, cars or for monitoring immobile patients in hospitals to prevent decubitus.

## 2. Experimental Section

### 2.1. Materials and Methods

All the polymers used were purchased from WackerChemie AG (München, Germany). The polymers were all from the Geniomer group and are copolymers containing a soft silicon part and a hard polyurethane part. Geniomer 100-HDS and Geniomer 100 consist of about 70% silicon and 30% polyurethane, while Geniomer 175 consists of only 10% polyurethane and 90% silicon and has a lower processing temperature than the two Geniomer 100 polymers. Geniomer 100-HDS is Geniomer 100 with the addition of highly dispersed silicate particles.

#### 2.1.1. Fibres Extrusion with Melt Flow Index

The transparent light pipes were produced by a melt flow index apparatus (MFI), model 7085.15a, provided by ZWICK (Ulm, Germany) with an external drawing engine. In Table 1 the extrusion parameters at which the fibres were extruded are shown. All tested materials were firstly dried in a vacuum oven for 8 h at 80 °C. 7 g were used for each test. Fibres made of Geniomer 100 were extruded with three different diameters. Since this material showed the best melt flow characteristics of all three polymers, the diameter of the light pipes could be easily varied with this polymer. Three different fibre diameters made of this material were studied: 0.45 mm, 0.75 mm, and 0.85 mm. Consequently, these optical fibres can support thousands of optical modes although the fibre does not feature an outer cladding to protect the surface from scratches or other defects.

#### 2.1.2. Light Loss

The optical loss measurements were performed with the cut-back method [22]. The medical laser used therefore had a wavelength of 652 nm with an intensity of 100 mW and was provided by AOL Medical Instruments. The 1 m long fibres were connected to the mode mixer (coupled to the light source) using F-SMA connectors from Thorlabs GmbH (Newton, MA, USA). The following equation was used to compute the light attenuation [23]. The optical loss measurements were repeated five times.

(1) $$A=10\cdot {\text{log}}_{10}\left(\frac{I}{I_{0}}\right)$$

where: *I* - Input light intensity, *I_0_*- Output light intensity.

**Figure 1.**
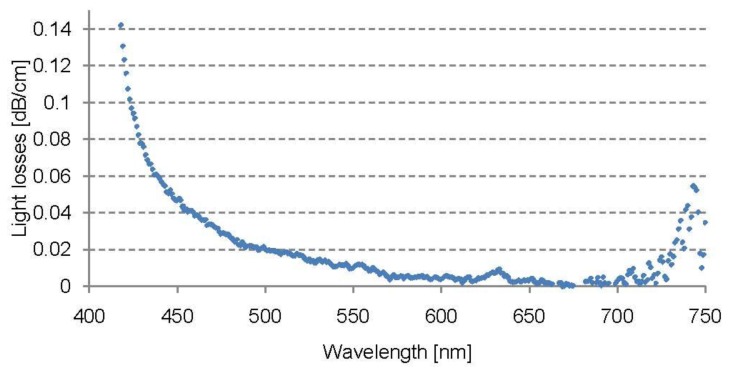
Intrinsic losses of the Geniomer 100-HDS polymer copolymer (measured from a polymer cube).

The optical loss measurements were repeated five times. Intrinsic losses over a whole visible spectrum range from 300 nm to 750 nm of Geniomer 100 were performed using a Lambda 900 UV-VIS spectrophotometer provided by Perkin Elmer. The spectrometer measures continuously and independently (by means of beam splitting) the intensity of the reference beam and uses the value to compute reflection or transmission coefficients. To determine the intrinsic loss of the copolymers (Figure 1), a polymer cube with the dimension 50 × 10 × 5 mm^3^ was formed. First the Fresnel reflection coefficient of the material was measured. This was done by measuring the light transmission through the thinnest part of the sample (5 mm), where the light absorption could be neglected. Afterwards, the light transmission through the 50 mm part was measured. The spectrophotometer was set to give the light transmission values from 300 nm to 750 nm in steps of 1 nm. For the force measurement setup, the extruded fibres were connected via a mode mixer to a Halogen-Deuterium Lamp model L10290 provided by Hamamatsu Photonics K.K. (Hamamatsu City, Japan), with SMA 905 connectors. In order to achieve flat fibre facets for good light coupling efficiency, fibres were cut with unused scalpel blades. Due to the elastic behaviour of the fibre material, polishing was not performed [17]. The ends of the fibres were connected by custom developed connectors to the measurement head RW3701-2 (Gigahertz Optik, Türkenfeld, Germany) and the outcoming light was measured using an Optometer P9710 detector (Gigahertz Optik). The force was applied with a Zwick INSTRON tensile testing machine model 4502 over the length of 3 cm. To study the influence of the temperature on the sensing system, the optical fibres were heated by a hot plate (Heidolph D-91126 type: MR Hei-Standard, Schwabach, Germany) and monitored by a temperature controller (Heidolph type: EHT Hei-Con). As a light source, an LED IFE97 and as a detector a photodiode IFD91 were used, both provided by Industrial Fiber Optics (Tempe, AZ, USA).

## 3. Results and Discussion

### 3.1. Optical Properties of the Extruded Optical Fibres

The force sensor is based on the losses in light transmission along the light guides when the fibres are compressed. Thus it was essential to check whether the extruded fibres have reasonable light transmission parameters, *i.e.*, low optical losses. Since the final goal is to incorporate the fibres to medical textiles with a max. length of 1 m, the maximum light attenuation should not exceed 0.2–0.3 dB/cm at 652 nm. Light losses at this level allow the detection of the transmitted light of a 1 m long light guide.

#### 3.1.1. Light Attenuation

In Table 2, a summary of light attenuation measurements at 652 nm is listed. No significant differences between the different materials and different diameters were observed. The optical signal loss varied between 0.16 and 0.25 dB/cm. That means that all fibres produced with the MFI apparatus were suitable for light transmissions over short distances of up to 1 m.

#### 3.1.2. Intrinsic Light Absorption Spectrum Measured at 652 nm

Figure 1 shows the intrinsic absorption spectrum of the light guide made of Geniomer 100-HDS as explained in Section 2.2. Since the overall attenuation of the light pipes was much higher (Table 2), the main light loss was due to extrinsic losses caused by the extrusion process (irregular fibre surface, bubbles or other inhomogeneities in the material). The absorption spectrum of Geniomer 100-HDS showed a local absorption band at around 635 nm, which corresponds to the 6th overtone of the C–H vibrations [24]. In the range of 660–700 nm, the absorption became lower than 0.01 dB/cm. Below 450 nm and above 700 nm the material starts to absorb strongly.

### 3.2. Force Sensing

**Figure 2.**
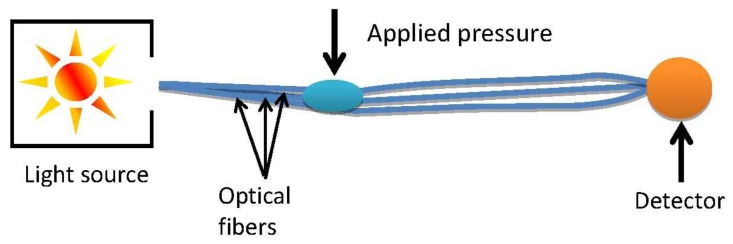
Force sensing setup.

The force sensing setup is presented below in Figure 2. The signal was measured from the middle of the three fibres. The outer fibres were used to support the weight.

**Figure 3.**
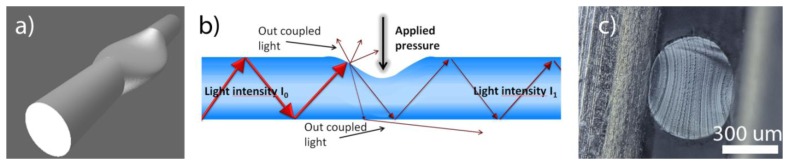
(**a**,**b**) Show schematically the influence of applied pressure on the elastic optical fibre. In (**c**) The cross-deformation of the fibre is demonstrated with a micrograph.

In Figure 3, a scheme representing the pressure sensing principle is shown. The applied force resulted in an elliptical deformation of the fibre cross section. From the side projection a kind of cavity was observed, as schematically illustrated in Figure 3. This deflection increased the out-coupling of the light in the region under pressure due to the geometrical deformation of the fibre. Since the degree of deflection was directly related to the applied force, the force could be quantitatively determined.

**Figure 4.**
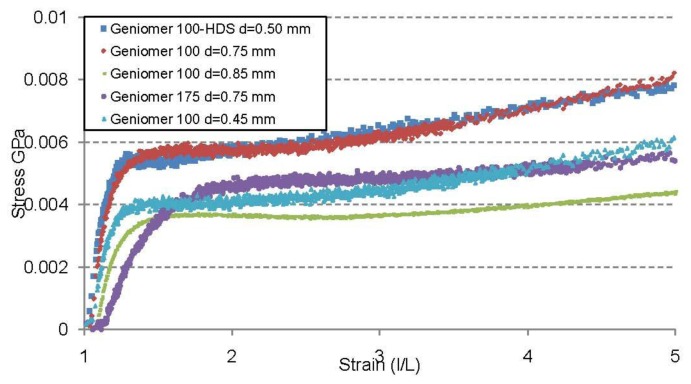
Stress strain curve of the different fibres produced in this study. The fibres made of Geniomer 175 show the lowest young's modulus.

From the strain-stress curve (Figure 4) it can be concluded that the fibre made of Geniomer 175 had a smaller elastic modulus than the fibres made of the other two Geniomer materials and thus should have the highest response for applied pressure. The Young moduli of Geniomer 100 and Geniomer 100-HDS are similar and big differences in the light transmission could not be observed while applying pressure. However, the strain-stress curve was measured along the fibres and not perpendicularly (as the force was applied) which can cause a discrepancy between young moduli and the drop in light transmission.

The force sensing experiments were conducted with the three different types of fibre materials. In order to check the repeatability, the measurements for each force were repeated three times. Each time the force was applied, it was completely released afterwards to check if the geometry of the fibre relaxed completely to its initial state. Firstly, the force of 1 N was applied three times to check whether the fibre was sensitive to the force at this level. The forces were then increased with increments of 10 N until the normalized signal output after releasing was below 90% of the original signal. If the signal did not recover 100%, the fibre was deformed not only elastically but also plastically. If the original shape was not recovered it was a sign that plastic deformation occurred [25]. The stress which is needed to start the plastic deformation is defined as the yield strength which was different for the three materials (Table 3).

Depending on the copolymer material used for extrusion, the applied forces caused different light transmission responses (Table 4). This was expected due to the different yield strengths shown in Table 3. Geniomer 100-HDS showed full reversibility up to 20 N and was therefore the material which was best suited for large forces. At the level of 30 N the signal went back to 95.8% ± 1.1%. Although the fibre did not relax completely at 30 N and 40 N, the measured signal after applying pressure was completely reproducible (σ = 0.0%) at these forces. Geniomer 100 and Geniomer 175 showed plastic deformation already at 10 N. Geniomer 100 did only reach 91% ± 2.5% of the initial signal after releasing from 10 N and was therefore the material with the lowest applicable forces for the sensing system. The sensitivity of the applied force on the fibre deflection is related to the young's modulus of the material. As expected from the strain-stress curve, the fibre made of Geniomer 175 had the highest response to applied force, as shown in Table 4. The drop in the signal of 20.8% ± 0.5% for 1 N was twice as much than the fibres produced from Geniomer 100 and Geniomer 100-HDS as presented in Table 4 (Sections 2 and 4). Due to the highest sensitivity of the fibres extruded of Geniomer 175, these fibres were chosen to measure the smallest detectable force. Forces ranging from 0.05 N to 0.5 N were applied three times (over 3 cm fibre length). From Section 1 in Table 4 it can be concluded that all the measured forces were successfully detected and the measurements were fully reproducible (σ = 0.0%). The smallest detectable force was as low as 0.05 N.

Since the light losses caused by the applied forces are associated to the fibre deflection, not only the polymer material but also the diameter of the fibre influenced the measurement range. This was demonstrated with the polymer Geniomer 100. By decreasing the fibre diameter, the fibres became more sensitive to the applied force. The fibre with the diameter of 0.85 mm was not sensitive enough at low forces (1 N) to get accurate and repeatable values as shown in Table 4. However, the thicker fibre showed a larger measurement window; forces of up to 40 N could be measured reversibly, while the thinnest fibre (0.45 mm) could only be used for forces of up to 10 N.

#### 3.2.1. Fibre Deflection against Signal Drop

**Figure 5.**
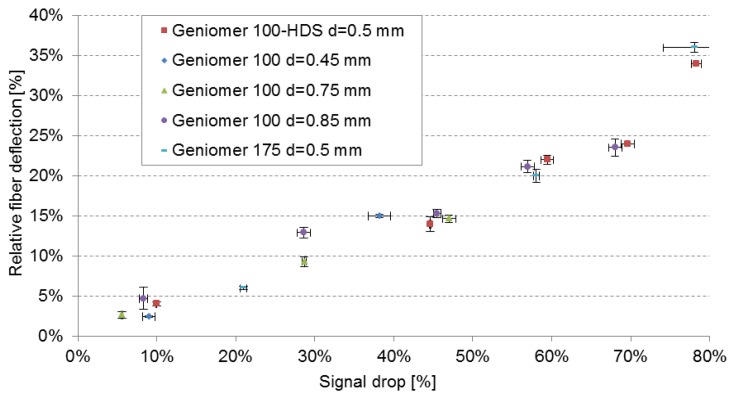
Fibre deflection *vs.* signal drop of fibres made of different materials and different diameters measured at 652 nm. Only the measurements with elastic deflections are shown.

In Figure 5 the relative fibre deflections in the force direction *vs.* the signal drop of all fibres (different polymers and different diameters) are presented. Only the measurements with strictly elastic deformations were included in the figure. The measurements of the fibre deflection were performed simultaneously with the force measurements using the tensile testing machine. Figure 5 clearly shows that the signal drop is correlated to the fibre deflection, independently of the polymer material and the fibre diameter. This shows that only the deflection in force direction influences the amount of the out-coupled light and that the elastic properties of the material has only a minor influence on the signal change. However, the sensitivity of the deflection towards the applied force is material dependent as shown above.

#### 3.2.2. Cross Sensitivity

Since this sensing technique relies on intensity measurements, it is essential to assess the influence of other parameters that can influence the light intensity. Below, we describe the influence of axial strain and bend losses on the fibre. The motion artifacts are not taken into account since they can be filtered out with signal processing.

**Figure 6.**
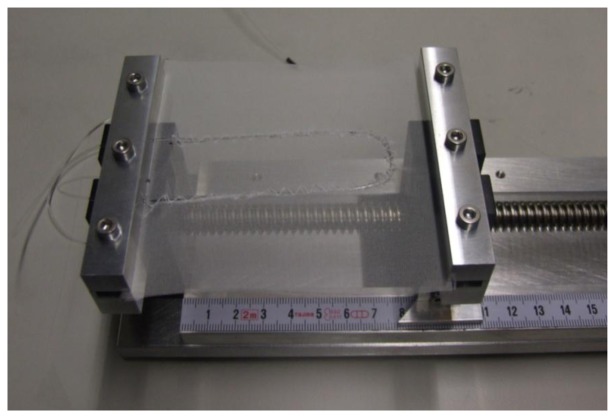
Measurement setup for axial strain influence.

**Figure 7.**
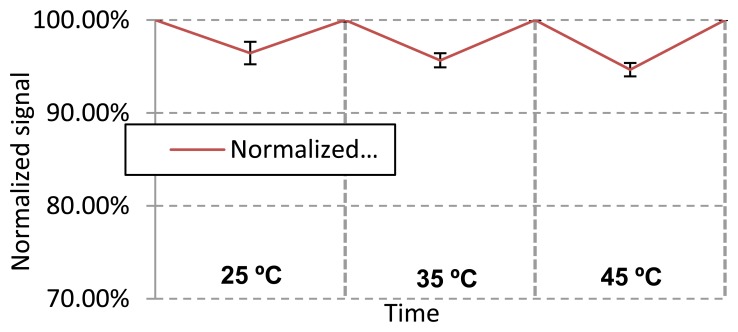
Signal dependency on the temperature. First, the attenuation was measured at a given temperature without applying any force. Then a force of 3 N was applied. The experiments were performed between 25 and 45 °C with 10 °C intervals and at a wavelength of 652 nm.

- Axial Strain Influence on the Sensor Signal
  In order to determinate whether the sensor is cross sensitive to axial strain, the fibre made of Geniomer 100 (d = 0.75 mm) was stitched into a woven textile (code name FRTT1022; provided by Forster Rohner, St. Gallen, Switzerland) as presented in Figure 6.
  According to the technical data sheet of this textile, the elastic deformation is 4%. The light intensity signal was measured simultaneously while the fabric was stretched with increments of 1.33%. In Table 5 the relative textile elongation *vs.* normalized signal is presented. It can be noticed that a loss in light transmission was detected when the fibre was stretched. At the maximum elongation of the fabric that was used, the signal dropped by 97% of the initial signal. This signal drop of 3% was lower than the signal drop of the smallest applied force in this study which was 1 N (signal drop of 5.7%). This leads us to the conclusion that axial strain would only influence the accuracy of the measurements at very low pressure. However, the influence of axial strain at low pressure can be bypassed by using a textile with a lower flexibility.
- Bend Losses
  To determine the influence of bend losses, the textile presented in Figure 5 was placed on three cylinders with radii 3, 5 and 6.5 cm respectively. Those numbers were chosen as the representative of human body parts: forearm, arm and thigh. In Table 6, the light loss *vs.* bend radius is presented. The signal when bended with radii 5 and 6.5 cm decreased less than 1%, which is most likely caused by applied pressure and contact with the cylinder. When the fabric was stronger bended with a radius of 3 cm, the light loss increased by up to ∼3.5%. The reason of the light loss was a combination of loss caused by increased imprinting of the matrix textile to the elastic optical fibre and of optical bend loss. This could be minimized by placing a cladding on the core of this waveguide, which in turn would protect the core from textile imprints. However, even at this level, the light loss was relatively small (detectable forces start at around 5.7% light loss).
  Table 6 shows the signal changes when a pressure was applied after the fibre was bent with a defined radius. As can be seen, the light loss was lower when the bending was narrower. The differences between the different radii became more significant when less force was applied.
- Temperature Dependency
  All the three Geniomer materials used have a Tg of around −130 °C and therefore have no phase change at higher temperature due to the amorphic nature of these polymers. In order to check the temperature dependence of the sensing properties, the experiments were performed under different temperatures. A force of 3 N was always applied to the Geniomer 100-HDS fibre with a diameter of 0.5 mm to show the reversibility at a certain temperature. Since this sensing system is supposed to be integrated into medical textiles in close contact with human beings, the measurements of the light losses with applied forces were performed at temperatures ranging from 25 to 45 °C with intervals of 10 °C (Figure 7). As can be seen in Figure 7, the temperature had no major influence on the light loss of the fibre while a force was applied.
- Inaccuracy
  Applied strain, bends and temperature change can influence the data. However, the accumulated errors should not exceed 10% of the signal change under the tested conditions. To determine for instance the breathing rate or to monitor the seat occupation, 10% of signal change would be acceptable. When detecting very low forces, as it would be the case for heart rate monitoring, the cross sensitivity becomes more important. Influences on the signal change have to be controlled by using other sensors (e.g., temperature sensor) or by controlling the environment of the fibre (e.g., avoiding of strain on the textile by using a non-elastic textile matrix). Since the optical power of LEDs is temperature-dependent, a control measurement may be necessary. The power fluctuation of the LED can be monitored by installing another LED of the same type for reference measurements into the measurement setup.

## 4. Conclusions

We demonstrate a simple way to manufacture force sensors based on light pipes in the form of multimode optical fibres made of copolymers. The working principle of the sensor is the use of the deflection of the fibre structure when a force is applied. By using materials with different young's modulus, the sensitivity of the material can be tuned according to the desired application in the range of 0.05–40 N over a fibre length of 3 cm. The fibres produced from Geniomer 175 are best suited for low forces, while the Geniomer 100-HDS fibres are ideal sensors for larger forces. Moreover, due to the flexibility and the high mechanical strength of the material, the proposed sensor can be easily integrated into textiles to form textile-based force sensors with possible applications as seat occupation monitoring in automotive or aeroplanes or as force sensors for medical applications (prevention for decubitus or breathing monitoring). Since the flexible fibres and not the electronics have to be placed on the measuring place, this sensing principle has no negative influence on the flexibility and the haptic of the textile. Only one wavelength is used for the measurement, which simplifies the electronic system consisting of an LED and a photo detector like e.g., a photodiode. With a thin polymer cladding with a lower refractive index than the copolymer waveguide core, the robustness of the sensor could be improved further. Scratches and imperfections at the fibre surface would then not deteriorate or mask the sensing signal.

## Figures and Tables

**Table 1.** Summary of parameters used for the fibre extrusions.

**Material type**	**Fibre Diameter** **[mm]**	**Temperature** **[°C]**	**Pre-worming** **time [s]**	**Load** **[kg]**	**Engine Speed** **[arbitrary units]**
Geniomer 100	0.45	163	240	1.2	5–6
Geniomer 100	0.75	160	240	2.16	10
Geniomer 100	0.85	165	240	3.8	2–3
Geniomer 175	0.5	160	240	2.16	10
Geniomer 100-HDS	0.5	160	240	2.16	10

**Table 2.** Summary of light attenuation measurements of the fibres produced in this study.

**Type of a Fibre**	**Light Attenuation [dB/cm]**	**σ^1^[dB/cm]**
Geniomer 100-HDS d = 0.5 mm	0.25	0.09
Geniomer 175 d = 0.5 mm	0.19	0.07
Geniomer 100 d = 0.75	0.22	0.06
Geniomer 100 d = 0.85 mm	0.20	0.09
Geniomer 100 d = 0.45 mm	0.24	0.12

1 Standard deviation.

**Table 3.** Yield strength of the extruded fibres.

**Copolymer Type**	**Yield Strength [GPa]**	**σ [GPa]**
Geniomer 100-HDS d = 0.5 mm	0.0054	0.0002
Geniomer 175 d = 0.5 mm	0.0045	0.0001
Geniomer 100 d = 0.45 mm	0.0039	0.0001

**Table 4.** Summary of force influence on light transmission.

	**Polymer type**	**Diameter** **[mm]**	**Force** **[N]**	**Normalized** **signal**	**σ**	**Normalized signal** **after releasing force**	**σ**
1	Geniomer 175	0.5	0.05	99.4%	0.1%	100.0%	0.0%
			0.1	98.8%	0.1%	100.0%	0.0%
			0.2	97.8%	0.0%	100.0%	0.0%
			0.5	94.3%	0.2%	100.0%	0.0%
			1	79.2%	0.5%	100.0%	0.0%
			10	42.0%	0.4%	100.0%	0.0%
			20	25.0%	0.7%	91.5%	1.7%
2	Geniomer 100	0.45	1	90.9%	1.0%	100.0%	0.0%
			10	61.6%	1.4%	91.0%	2.5%
3	Geniomer 100-HDS	0.5	1	90.0%	0.3%	100.0%	0.0%
			10	55.1%	0.6%	100.0%	0.0%
			20	40.6%	0.5%	100.0%	0.0%
			30	30.4%	0.0%	95.8%	1.1%
			40	21.7%	0.0%	90.6%	0.9%
4	Geniomer 100	0.75	1	94.3%	0.2%	100.0%	0.0%
			10	71.3%	0.6%	100.0%	0.0%
			20	53.0%	0.5%	90.7%	0.3%
5	Geniomer 100	0.85	1	91.7%	1.4%	100.0%	0.0%
			10	71.4%	0.7%	100.0%	0.0%
			20	54.5%	0.5%	100.0%	0.0%
			30	43.1%	0.8%	95.6%	1.0%
			40	31.9%	1.1%	91.7%	0.9%

**Table 5.** Strain influence on the light transmission trough the sensor.

**Relative Textile Elongation**	**Normalized Signal**
0.00%	100.0%
1.33%	99.6%
2.67%	99.0%
4.00%	97.0%

**Table 6.** Influence of fibre bendings on the relative light loss.

**Bending** **Radius** **[cm]**	**Light Loss** **by Bending**	**σ**	**Light Loss when 1** **N** **Force Applied**	**σ**	**Light Loss when** **20 N Force** **Applied**	**σ**
3	3.43%	0.24%	5%	0.4%	43.1%	0.2%
5	0.91%	0.26%	7.9%	0.4%	44.6%	0.3%
6.5	0.89%	0.93%	8.8%	0.4%	45.1%	0.6%
